# Response to pregabalin and progesterone differs in male and female rat models of neuropathic and cancer pain

**DOI:** 10.1080/24740527.2020.1724776

**Published:** 2020-02-28

**Authors:** Robert G. Ungard, Yong Fang Zhu, Sarah Yang, Peter Nakhla, Natalka Parzei, Kan Lun Zhu, Gurmit Singh

**Affiliations:** a Michael G. DeGroote Institute for Pain Research and Care, Medicine, McMaster University, Hamilton, Ontario, Canada; b Department of Pathology and Molecular Medicine, McMaster University, Hamilton, Ontario, Canada

**Keywords:** cancer pain, neuropathic pain, electrophysiology, sensory neurons, dorsal root ganglion, behavior, pregabalin, progesterone

## Abstract

**Background**: Cancer pain involves nervous system damage and pathological neurogenesis. Neuropathic pain arises from damage to the nervous system and is driven by ectopic signaling. Both progesterone and pregabalin are neuroprotective in animal models, and there is evidence that both drugs bind to and inhibit voltage-gated calcium channels.

**Aims**: This study was designed to characterize the effects of progesterone and pregabalin in preclinical models of cancer and neuropathic pain in both sexes.

**Methods**: We measured peripheral sensory signaling by intracellular in vivo electrophysiology and behavioral indicators of pain in rat models of cancer-induced bone pain and neuropathic pain.

**Results**: Female but not male models of cancer pain showed a behavioral response to treatment and pregabalin reduced excitability in C and A high-threshold but not low-threshold sensory neurons of both sexes. Male models of neuropathic pain treated with pregabalin demonstrated higher signaling thresholds only in A high-threshold neurons, and behavioral data indicated a clear recovery to baseline mechanical withdrawal thresholds in all treatment groups. Female rat treatment groups did not show excitability changes in sensory neurons, but all demonstrated higher mechanical withdrawal thresholds than vehicle-treated females, although not to baseline levels. Athymic female rat models of neuropathic pain showed no behavioral or electrophysiological responses to treatment.

**Conclusions**: Both pregabalin and progesterone showed evidence of efficacy in male models of neuropathic pain. These results add to the evidence demonstrating differential effects of treatments for pain in male and female animals and widely differing responses in models of cancer and neuropathic pain.

## RÉSUMÉ

**Contexte**: La douleur cancéreuse implique des lésions du système nerveux et une neurogenèse pathologique. La douleur neuropathique résulte d'une lésion du système nerveux et est provoquée par une signalisation ectopique. La progestérone et la prégabaline sont toutes deux neuroprotectrices dans les modèles animaux et il est prouvé que ces deux médicaments se lient aux canaux calciques à tension contrôlée et les inhibent.

**Objectifs**: Cette étude visait à caractériser les effets de la progestérone et de la prégabaline dans des modèles précliniques de cancer et de douleur neuropathique chez les deux sexes.

**Méthodes**: Nous avons mesuré la signalisation sensorielle périphérique par électrophysiologie intracellulaire in vivo, ainsi que les indicateurs comportementaux de la douleur dans des modèles de rats atteints de douleurs osseuses et de douleurs neuropathiques induites par le cancer.

**Résultats**: Contrairement aux modèles masculins, les modèles féminins atteints de douleur cancéreuse ont montré une réponse comportementale au traitement, tandis que la prégabaline a réduit l'excitabilité des neurones sensoriels C et A à seuil élevé mais non à seuil bas chez les deux sexes. Les modèles masculins atteints de douleur neuropathique traités à la prégabaline ont montré des seuils de signalisation plus élevés uniquement dans les neurones A à seuil élevé, tandis que les données comportementales ont indiqué un net retour aux seuils de retrait mécanique de départ dans tous les groupes de traitement. Les groupes de rats femelles traités n'ont pas montré de changements d'excitabilité dans les neurones sensoriels, mais tous ont montré des seuils de retrait mécanique plus élevés que les femelles traitées avec le vecteur, sans toutefois atteindre les niveaux de départ. Les modèles atteints de douleur neuropathique parmi les rats femelles athymiques n'ont montré aucune réponse comportementale ou électrophysiologique au traitement.

**Conclusions**: La prégabaline et la progestérone ont toutes deux démontré leur efficacité dans les modèles masculins atteints de douleur neuropathique. Ces résultats s'ajoutent aux données probantes démontrant les effets différentiels des traitements de la douleur chez les animaux mâles et femelles, et les réponses très différentes dans les modèles atteints de cancer et de douleur neuropathique.

## Introduction

Neuropathic pain is a prevalent and often intractable state of pain arising from pathology of the peripheral or central nervous system and driven by ectopic signaling from damaged or pathological neurons.^1,2^ Cancer pain is also often severe and intractable and can be induced by multiple stimuli. These include nociceptive mechanical and chemical stimuli that result from the cancer growth and metastasis to the bone, as well as damage to and pathology of the nervous system itself. As a result, cancer pain is described as a unique pain state that includes aspects of nociceptive, neuropathic, and inflammatory pain.^3,4^

In models of neuropathic pain (NEP), treatment with progesterone (PRO) has produced beneficial results, including restoring myelination of damaged neurons and ameliorating mechanical and thermal withdrawal thresholds in animal models of nerve crush,^5^ spinal cord injury,^6^ and chemotherapy-induced NEP.^7^ In a rat model of sciatic cuff–induced NEP, PRO treatment starting immediately after model induction and lasting for 10 days significantly limited the development of mechanical allodynia.^8^ Early clinical reporting describes treatment with PRO sharply reducing or abolishing pain in patients with late-stage metastatic breast cancer including bone metastases.^9^ However, PRO has yet to be investigated in animal models of cancer pain.

Calcium signaling plays a well-established role in neuronal inflammation, demyelination, and excitotoxic cell death,^10^ all of which are involved in the generation and maintenance of NEP and which progesterone treatment has been experimentally demonstrated to reduce. In cultured rat striatal neurons, supraphysiological doses of PRO have been demonstrated to inhibit excitotoxic neuronal cell death by direct inhibition of L-type voltage-gated calcium channel (VGCC) action, without effect on glutamate-mediated ion channels.^11^

Pregabalin (PRE) is a well-established anticonvulsant and analgesic drug approved for the management of NEP associated with diabetic peripheral neuropathy and postherpetic neuralgia. PRE also demonstrates clinical utility across many other NEP conditions, including chemotherapy-induced peripheral neuropathy, trigeminal neuralgia, fibromyalgia, and postsurgical pain.^12^ PRE is utilized to treat neuropathic cancer pain in the clinic. Some clinical studies demonstrate its utility in comparison to other drugs,^13,14^ whereas other studies find no beneficial effects.^15^ Despite these discrepancies, PRE has not been tested in animal models of cancer pain excluding chemotherapy-induced neuropathies. Similarly to PRO, PRE has been shown to act as a VGCC antagonist by binding at the α2-δ auxiliary subunits of P/Q-, N-, and L-type VGCCs.^16^ This VGCC inhibition and the resulting reduction of Ca^2+^-mediated excitatory glutamate release at neuronal synapses confers a neuroprotective benefit^17^ and is thought to be responsible for the effectiveness of PRE in treating NEP.^12^

Due to the evidence of neuropathic involvement in cancer-induced bone pain (CIBP) and the widespread evidence of efficacy of the VGCC antagonists PRO and PRE in conditions of NEP, this investigation was designed to characterize the behavioral and electrophysiological effects of these drugs in a rat model of CIBP. Our findings are indicative of a limited and possibly sexually divergent response in CIBP and prompted further investigation in our well-established rat model of sciatic cuff–induced neuropathy.

## Materials and Methods

### Cell Culture

The mammary rat metastasis tumor (MRMT-1) rat mammary carcinoma cell line (provided by Dr. Philippe Sarret of the Université de Sherbrooke, Sherbrooke, QC, Canada) was used in all in vitro and in vivo work. Cells were maintained in a humidified incubator at 37°C with 5% CO_2_ in growth medium supplemented with 10% fetal bovine serum (FBS) and antibiotics (100 U ml^−1^ penicillin sodium and 1% antibiotic/antimycotic (Thermo Fisher Scientific, Inc., Waltham, MA). MRMT-1 cells were grown in RPMI 1640 (Thermo Fisher Scientific) and tested for mycoplasma contamination prior to experimental use. Cell numbers were quantified in 96-well plates using crystal violet staining, measuring absorbance at λ = 570 nm with an optical plate reader (BioTek, Winooski VT). Cells treated with PRE were plated with dialyzed FBS, and cells treated with PRO were plated with charcoal-stripped FBS (Thermo Fisher Scientific) for all measurements. All were plated at 8000 cells/well and measured 24 h posttreatment. Cell numbers are indicated relative to their respective vehicle-only control for each dose. Cell harvesting for in vivo implantation was performed on subconfluent cultures; adherent cells were suspended and kept lightly agitated in sterile Hank’s Balanced Salt Solution on ice.

### Test Compounds

Progesterone (4-pregnene-3,20-dione; Sigma-Aldrich, Oakville, ON, Canada) was administered in vivo at 30 mg/kg, suspended in sesame oil. Pregabalin was administered at 4 mg/kg dissolved in 0.9% saline. Earlier experimentation has demonstrated the doses included here to be relevant in animal models of pain and within published safe dosing ranges.^8,12^ Vehicle-treated controls (0.9% saline) were tested in parallel with experimental animals. Drug solutions were freshly prepared and administered by daily intraperitoneal injection.

### Animal Models

All procedures were conducted according to the guidelines of the Committee for Research and Ethical Issues of the International Association for the Study of Pain^18^ and guidelines established by the Canadian Council on Animal Care with ethical approval from the McMaster University Animal Research Ethics Board. All experimental animals were housed in pairs with access to food and water ad libitum in a temperature-controlled room under a 12-h light:dark cycle.

### Cancer Pain Models

Male and female Sprague-Dawley (SD) rats (Charles River Inc., Saint-Constant, QC, Canada) weighing 170–200 g were utilized for all cancer models. Rats were randomly assigned to cancer or sham surgery groups. MRMT-1 cells (3.0 × 104) resuspended in 20 μL Hank’s Balanced Salt Solution were implanted in the distal femur of each cancer pain model rat. Cells for sham surgical controls were suspended at the same concentration and inactivated by three heat:freeze cycles prior to implantation.

Rats were anaesthetized with inhaled isoflurane (3%–5% in O_2_) and oriented in a supine position with their right hind limb fixed to a stationary convex support to maintain the limb in a flexed position. A small incision was made on the medial side to expose the quadriceps femoris and the vastus lateralis was incised to expose the medial epicondyle of the femur. A small cavity was drilled between the medial epicondyle and the adductor tubercle with a 0.8 A stereotaxic drill equipped with a 1.75-mm burr. A 25-gauge needle was inserted into this cavity to penetrate the intramedullary canal. The needle was removed and replaced with a blunted 25-gauge needle attached to a Hamilton syringe containing the live MRMT-1 or heat/freeze-inactivated MRMT-1 (sham) cell suspension. The suspension was dispensed slowly into the canal and the syringe was left in place for 1 min to prevent leakage. The cavity was then sealed with dental amalgam and fixed using a curing light. The wound was flushed with sterile deionized water, and muscle, fascia, and skin were sutured. Cancer cell implantation to the distal femur was performed as described in detail in previously published methods.^19,20^

### Neuropathic Pain Models

Male and female SD rats and female RNU^−/−^ immunocompromised rats weighing 170–200 g were used for all NEP models. A peripheral neuropathy was induced by the “sciatic cuff model” according to methods first described by Mosconi and Kruger^21^ and described in detail in previously published work.^22^ Animals were anesthetized with a mixture of ketamine (Narketan; 5 mg/100 g; Vetoquinol N.-A. Inc., Lavaltrie, QC, Canada), xylazine (Rompun; 0.5 mg/100 g; Bayer Inc., Toronto, ON, Canada), and acepromazine (Atravet; 0.1 mg/100 g; Ayerst Veterinary Laboratories, Guelph, ON, Canada) given intraperitoneally, and the right sciatic nerve was exposed at the mid-thigh level. One cuff of 0.5-mm polyethylene (PE-90) tubing (Intramedic PE-90, Fisher Scientific Ltd., Whitby, ON, Canada) was slit longitudinally and fitted around the exposed nerve. The muscle and skin of the wound were then sutured separately.

### Behavioral Analyse*s*

Rats were exposed to handling and behavioral testing equipment for a 1- to 2-week acclimation period and assigned individual identification prior to model induction. All behavioral testing was repeatedly performed by the same operators, who were blinded to group assignment throughout the duration of the study. Behavioral testing was performed prior to model induction to obtain baseline data and weekly beginning on day 7 following model induction, continuing until endpoints, which were week 3 post model induction for all CIBP models and week 6 post model induction for all NEP models. With the exception of week 1 acute testing, all behavior measurements were performed prior to daily drug administration.

### Dynamic Weight Bearing

Weight, area, and time distribution between all points of pressure of freely moving animals were recorded with the Dynamic Weight Bearing test 2.0 (DWB) (BioSeb, Vitrolles, France). Each animal was recorded in the DWB apparatus for 5 min/test and recordings were manually validated with DWB software version 2.0.59 (BioSeb). Results were exported as mean weight and time for each point of pressure across the validated experiment time. DWB has been validated as a useful test for animal models of CIBP.^20,23^ Postural disequilibrium of the animal could indicate an allodynic response to normal ambulation, so a reduction in weight borne by the tumor-afflicted limb of the animal was accepted as evidence of an inability or aversion to utilize that limb, providing indirect evidence of nociception.

### Limb Use Scale

The open field observational limb use scale is an operator-derived numerical representation of the use of the animal’s ipsilateral limb, scored over a 5-min period of free ambulation (0 = *no use*, 1 = *severe limp*, 2 = *moderate limp*, 3 = *slight limp*, 4 = *normal use*). This scale has been validated in mouse models of cancer-induced bone pain.^24,25^

### von Frey Mechanical Withdrawal

To quantify mechanical sensitivity, brisk foot withdrawal in response to normally innocuous mechanical stimuli with von Frey filaments was measured. Rats were placed in a 30 × 30 × 30 cm Plexiglas box designed for von Frey testing with a clear floor containing 0.5-cm-diameter holes spaced 1.5 cm apart for access to the paws.^26^ Rats were habituated to the box for a minimum of 15 min until cage exploration and major grooming activities ceased, prior to any stimulation. Von Frey filaments (Stoelting Co., Wood Dale, IL) were applied to the plantar surface of the ipsilateral hind paw to determine mechanical withdrawal thresholds using the up–down method of Dixon^27^ as applied to rodents by Chaplan et al.^28^ A von Frey filament was applied a maximum of five times for 3–4 s each, at 3-s intervals, to different spots on the plantar surface of the ipsilateral hind paw in ascending order of force until a clear withdrawal response was observed, starting with the 2 g filament. When a withdrawal occurred, the next lightest filament was reapplied, and the process continued until a 50% withdrawal response threshold was derived. Brisk foot withdrawal in response to the mechanical stimulus was interpreted as a valid response. A reduction in 50% mechanical withdrawal threshold by the tumor- or cuff-afflicted limb was indicative of allodynia.

### Intracellular in vivo Electrophysiology

Details of intracellular electrophysiological recording techniques have been reported previously in animal models of NEP^2,22,29^ and cancer pain.^30,31^ Briefly, action potentials evoked by stimulation of the dorsal root and measured at the L4 DRG soma are used to compare the configuration parameters and conduction velocity of each neuron. Recorded neurons were classified as C-type high-threshold mechanosensitive fibers (CHTM), Aβ-type high-threshold mechanosensitive fibers (AHTM), or Aβ-low-threshold mechanosensitive fibers (ALTM) based on their action potential configuration, conduction velocity, and receptive field properties as determined by utilizing handheld mechanical stimulators.^22,32,33^ Other major factors, including the rate of adaptation and the tissue location of the receptive field, were used to further classify ALTM neurons as either cutaneous neurons (CUT) or muscle spindle (MS) neurons. MS neurons were classified as slowly adapting neurons with deep subcutaneous receptive fields activated by deep tissue manipulation of the muscle belly but not by cutaneous stimulation.

Soma excitability thresholds were measured by evoking action potentials in the somata of DRG neurons by direct injection of depolarizing current. To quantify soma excitability, current injections of 100 ms each were injected into the soma, at amplitudes between 0.5 to 4 nA in increments of 0.5 nA. The thresholds of depolarizing current pulses were determined with the “Protocol Editor” function in the pClamp 9.2 software program (Molecular Devices). All animals were tested at model endpoint. CIBP model rats were recorded following week 3 behavioral testing and all NEP model rats were recorded following week 6 behavioral testing.

### Statistical Analyses

In vitro data represent the mean of *n* = 3 biological replicates plus or minus the standard error of the mean (SEM). Data are expressed as fold change relative to dose-matched vehicle controls. Differences within treatment groups are compared to untreated control (0 µM) by one-way analysis of variance (ANOVA) with post hoc Dunnett’s multiple comparisons test. In vivo behavioral data represent the mean ± SEM from male SD CIBP rats (PRE *n* = 6, PRO *n* = 5, vehicle *n* = 5, sham *n* = 5) and female SD CIBP rats (PRE *n* = 6, PRO *n* = 4, vehicle *n* = 6, sham *n* = 7). Only animals with verified tumor development are included in results for CIBP model animals. Data were collected from male SD NEP rats (PRE *n* = 4, PRO *n* = 4, PRE+PRO *n* = 4, vehicle *n* = 4), female SD NEP rats (PRE *n* = 6, PRO *n* = 6, PRE+PRO *n* = 4, vehicle *n* = 4 rats), and female RNU^−/−^ NEP rats (PRE *n* = 5, PRO *n* = 5, vehicle *n* = 4, Naïve *n* = 3). All behavioral results are compared between and within groups across the duration of the experiment, and groups are compared independently at endpoint. Differences between treatment groups over time are compared to vehicle control by repeated measures two-way ANOVA with post hoc Dunnett’s multiple comparisons test. Differences within treatment groups relative to respective baseline measurements are compared by repeated measures two-way ANOVA with post hoc Dunnett’s multiple comparisons test. Differences between treatment groups at endpoint are compared by one-way ANOVA with post hoc Tukey’s multiple comparisons test or Kruskal-Wallis test with post hoc Dunn’s multiple comparisons test for nonparametric limb use scale data. Acute response differences within treatment groups are compared by multiple *t*-tests. Electrophysiological data represent the mean ± SEM from independently recorded neurons from *n* ≥ 3 rats of each group. Differences between treatment groups are compared by Kruskal-Wallis test with post hoc Dunn’s multiple comparison test. All results were considered significant at *P* < 0.05. Analyses and charts were generated using GraphPad Prism 7 software (GraphPad Software, La Jolla, CA).

## Results

### Pregabalin and progesterone treatment delays the onset of mechanical hypersensitivity relative to vehicle treatment in female rat models of cancer-induced bone pain but have no effects in males

**Figure 1.**
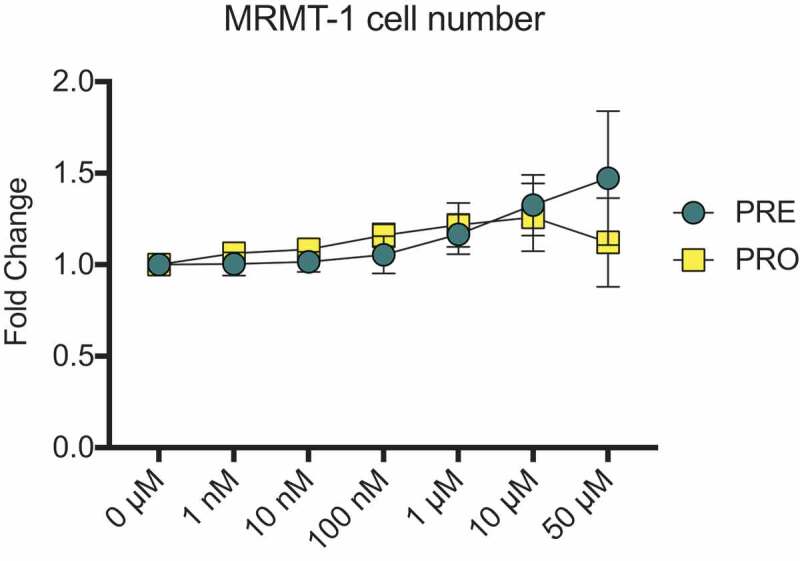
MRMT-1 cell number is unaffected by treatment with pregabalin or progesterone

Prior to use in animal models, the growth of MRMT-1 cancer cells treated with PRE and PRO was investigated in vitro to determine whether administration of these test compounds could affect cell growth and therefore tumor size and nociceptive outcomes in in vivo animal models. Cell number was measured in vitro in the presence of a range of PRE and PRO doses between 1 nM and 50 µM. Crystal violet staining showed no differences between vehicle-treated MRMT-1 cells and PRE- and PRO-treated cells at any dose (Figure 1). Due to these findings, no differences were expected to occur in bone tumor size between treated and untreated rats due to direct effects of treatment compounds on cancer cells.

Crystal violet stain for cell number showed no differences between vehicle-treated MRMT-1 cells and PRE- and PRO-treated cells at doses ranging between 1 nM and 50 µM. This in vitro assay was to determine whether the experimental drugs may affect tumor growth in our in vivo models of cancer pain. Each treatment dose is expressed as fold change relative to the respective vehicle at that same dose, PRO was suspended in sesame oil; PRE was suspended in saline. Data represent the mean of *n* = 3 biological replicates ± SEM. Differences within treatment groups were compared to untreated control (0 µM) by one-way ANOVA with post hoc Dunnett’s test.

**Figure 2.**
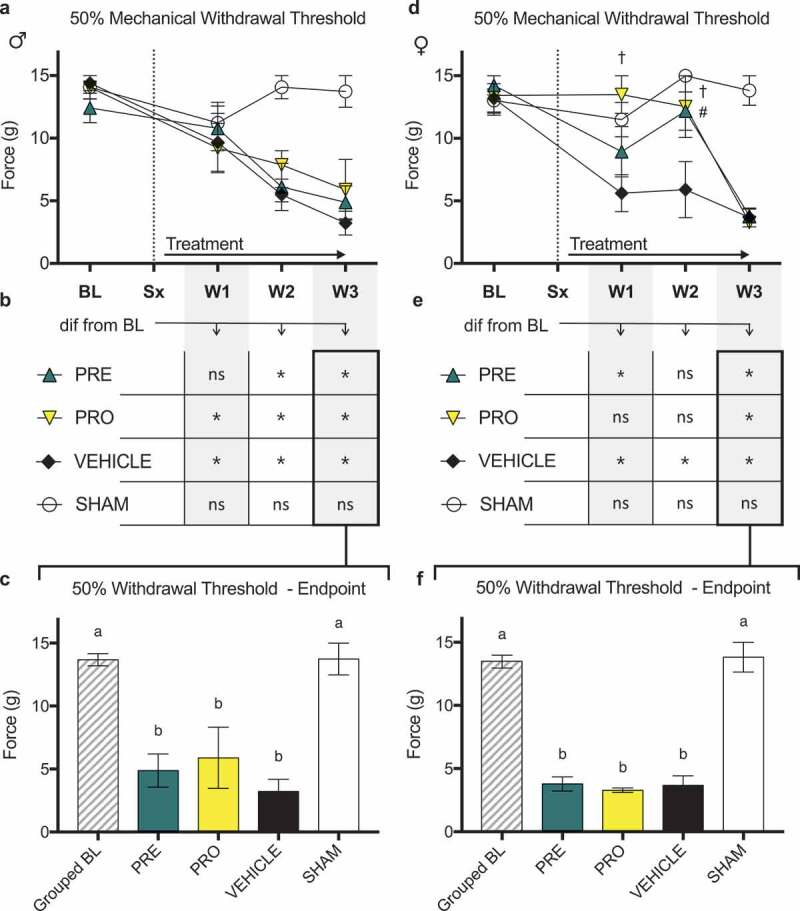
Both pregabalin- and progesterone-treated female rat models of cancer-induced bone pain showed a delay until the onset of a reduced mechanical withdrawal threshold relative to vehicle-treated rats. Male rats showed no differences between treatment groups

Male CIBP model rats did not show sustained differences indicative of reduced nociception by any behavioral measures in PRE- or PRO-treated groups when compared to vehicle. There were no differences in 50% mechanical paw withdrawal threshold in the ipsilateral limb between PRO, PRE, and vehicle treatment groups of male CIBP model rats at any time point post model induction as measured by testing with von Frey filaments (Figure 2a). All male model groups showed reduced thresholds relative to sham surgical control animals in weeks 2 and 3. Likewise, no treatment groups of male rats including vehicle treatment showed a sustained delay past week 1 until the mechanical withdrawal threshold was reduced relative to the baseline measurements for each group (Figure 2b). At endpoint (Figure 2c), all treatment groups including vehicle were not different from each other and all showed significantly decreased paw withdrawal thresholds relative to baseline and to sham control thresholds.

(a) There were no differences in 50% mechanical paw withdrawal threshold in the ipsilateral limb between PRO, PRE, and vehicle treatment groups of male CIBP model rats at any time point post model induction as measured by testing with von Frey filaments. All male model groups showed reduced thresholds relative to sham surgical control animals in weeks 2 and 3 (not marked on chart). (b) Likewise, no treatment groups of male rats showed a sustained delay past week 1 until the withdrawal threshold was reduced relative to baseline measurements. (c) All treatment groups were not different from each other and all showed significantly decreased paw withdrawal thresholds relative to baseline and to sham control thresholds at endpoint. (d) Female CIBP model rats showed evidence of a delay of onset of a reduced mechanical withdrawal threshold in both PRO- and PRE-treated groups relative to vehicle, where PRE-treated animals were significantly different from vehicle at week 2 and PRO-treated animals showed higher thresholds at both weeks 1 and 2. No groups are different at week 3. (e) Similarly, PRE-treated animals were not different from their respective baseline measurements at week 2, and PRO-treated animals were not different from baseline until week 3. Vehicle-treated animals were different from baseline at each week following tumor implantation, and sham animals did not differ from baseline at any time point. (f) At endpoint there were no differences between treatment groups and all showed significantly decreased paw withdrawal thresholds relative to baseline and to sham control.Data represent the mean ± SEM from male SD rats (PRE, *n* = 6; PRO, *n* = 5; vehicle, *n* = 5; sham, *n* = 5) and female SD rats (PRE, *n* = 6; PRO, *n* = 4; vehicle, *n* = 6; sham *n* = 7). Only animals with verified tumor development were included in results shown. Differences between treatment groups over time (a, d) are compared to vehicle control by repeated measures two-way ANOVA with post hoc Dunnett’s test (#*P* < 0.05, PRE vs. vehicle; †*P* < 0.05, PRO vs. vehicle). Differences within treatment groups relative to baseline measurements (b, e) are compared by repeated measures two-way ANOVA with post hoc Dunnett’s test (*P < 0.05). Differences between treatment groups at endpoint (c, f) are compared by one-way ANOVA with post hoc Tukey’s test. Different letters (a, b) represent differences between groups (*P* < 0.05).

Female CIBP model rats showed evidence of a delay of onset of a reduced mechanical withdrawal threshold in both PRO- and PRE-treated groups relative to vehicle (Figure 2d), where PRE-treated animals were significantly different from vehicle at week 2 and PRO-treated animals showed higher thresholds in both weeks 1 and 2. No groups were different at week 3. Consistently, PRE-treated animals did not respond at lower mechanical force from baseline measurements at week 2 (Figure 2e), and PRO-treated animals did not react to lower stimuli from their respective baselines until week 3. Vehicle-treated animals reacted to less force than baseline at each week following tumor implantation, and sham animals did not differ from baseline measurements at any time point. At endpoint (Figure 2f), there were no differences between treatment groups and all showed significantly decreased paw withdrawal thresholds relative to baseline and to sham control.

**Figure 3.**
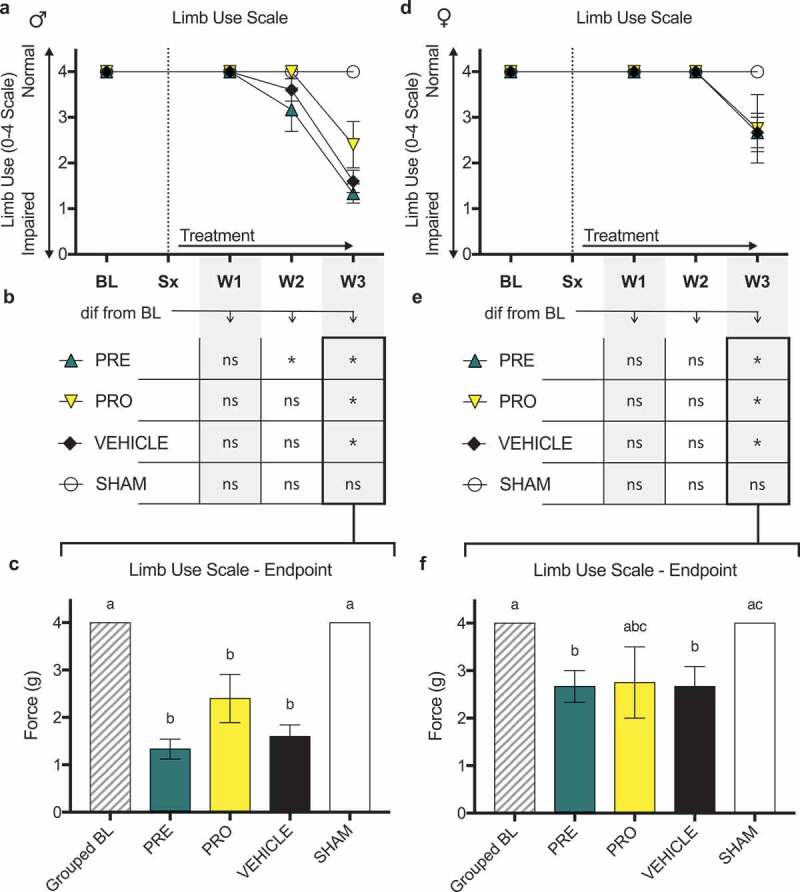
No female or male rat models of cancer-induced bone pain showed differences in limb use between treatment groups or vehicle treatment as measured by observational scoring

There were no differences in ipsilateral limb use scoring (0–4 scale) as measured by observational scoring over a 5-min period of free ambulation between PRO, PRE, and vehicle treatment groups of (Figure 3a) male and (Figure 3d) female CIBP model rats at any time point post model induction. All male and female groups showed impaired limb use relative to sham surgical control animals by week 3. Likewise, no treatment groups, including vehicle, of male (Figure 3b) and female (Figure 3e) rats showed sustained maintenance of normal limb use past week 2 relative to their respective baseline measurements. At week 3 all treatment groups of male rats showed significantly decreased limb use scores relative to baseline and to sham controls (Figure 3c) and all treatment groups including vehicle were not different from each other. At endpoint, female PRE- and vehicle-treated groups were significantly decreased from baseline and sham, whereas PRO-treated rats showed no differences from any groups (mean ± SEM: PRE, 2.67 ± 0.33; vehicle, 2.67 ± 0.42; PRO, 2.75 ± 0.75; sham, 4 ± 0; Figure 3f).

There were no differences in ipsilateral limb use scoring (0–4 scale) between PRO, PRE, and vehicle treatment groups of (a) male and (d) female CIBP model rats at any time point post model induction as measured by observational scoring over a 5-min period of free ambulation. All male and female groups showed impaired limb use relative to sham surgical control animals by week 3 (not indicated on charts). Likewise, no treatment groups including vehicle of (b) male and (e) female rats showed sustained maintenance of normal limb use past week 2 relative to baseline measurements. (c) At week 3 endpoint, all treatment groups of male rats were not different from each other and all showed significantly decreased limb use scores relative to baseline and to sham controls. (f) At endpoint, female PRE- and vehicle-treated groups were significantly decreased from baseline and sham, whereas PRO-treated rats showed no differences from any groups.Data represent the mean ± SEM from male SD rats (PRE, *n* = 6; PRO, *n* = 5; vehicle, *n* = 5; sham, *n* = 5) and female SD rats (PRE, *n* = 6; PRO, *n* = 4; vehicle, *n* = 6; sham, *n* = 7). Only animals with verified tumor development were included in results shown. Differences between treatment groups over time (a, d) are compared to vehicle control by repeated measures two-way ANOVA with post hoc Dunnett’s test (*P* < 0.05). Differences within treatment groups relative to baseline measurements (b, e) are compared by repeated measures two-way ANOVA with post hoc Dunnett’s test (*P < 0.05). Differences between treatment groups at endpoint (c, f) are compared by Kruskal-Wallis test with post hoc Dunn’s test. Different letters (a, b, c) represent differences between groups (*P* < 0.05).

**Figure 4.**
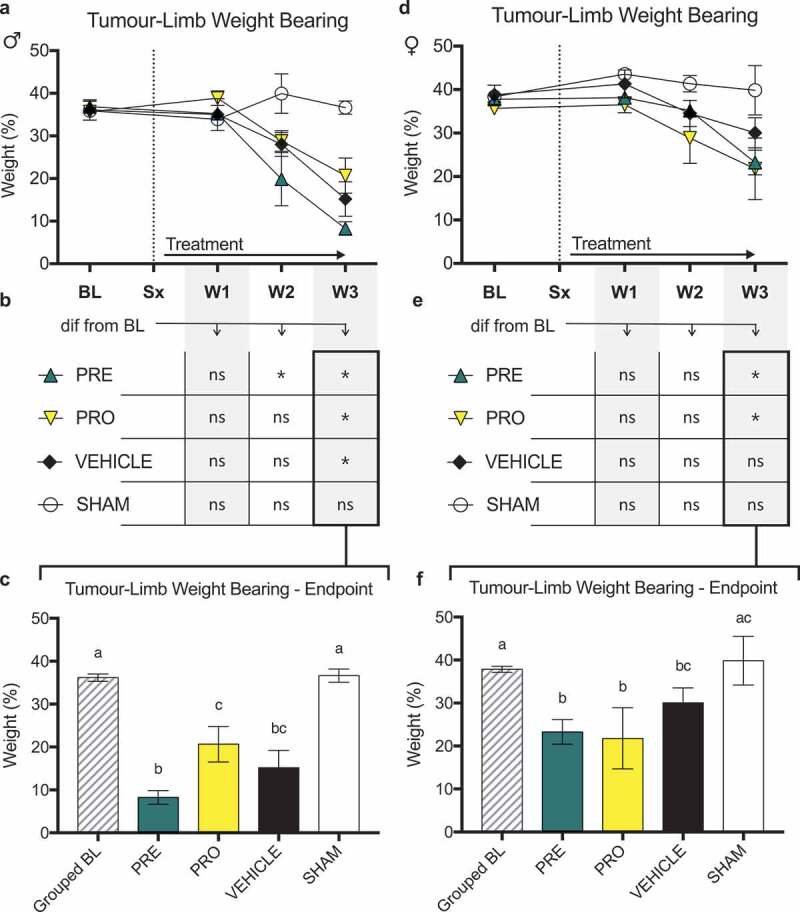
No female or male rat models of cancer-induced bone pain showed differences in ipsilateral limb weight bearing between treatment groups and vehicle treatment

There were no differences in ipsilateral limb weight bearing as a percentage of animal body weight as measured by dynamic weight bearing between PRO, PRE, and vehicle treatment groups of male (Figure 4a) and female (Figure 4d) CIBP model rats at any time point post model induction. No treatment groups of male rats (Figure 4b) showed sustained maintenance of normal ipsilateral weight bearing past week 2 relative to baseline measurements of each group. PRE- and PRO-treated female rats also showed decreased weight bearing at week 3; however, vehicle-treated rats were not different from their respective baseline measurements. At week 3 endpoint all treatment groups of male rats showed significantly decreased ipsilateral limb weight bearing relative to baseline and to sham controls (Figure 4c), and PRE-treated male rats were significantly lower than PRO-treated male rats. At endpoint, female PRE- and PRO-treated groups were significantly decreased from baseline and sham (Figure 4f); however, vehicle-treated rats were significantly decreased from grouped baseline measurements only and not different from PRE or PRO.

### High- but not low-threshold mechanosensitive fibers in CIBP model animals treated with PRE have excitability thresholds that are higher than those of vehicle and equivalent to those of sham controls.

**Figure 5.**
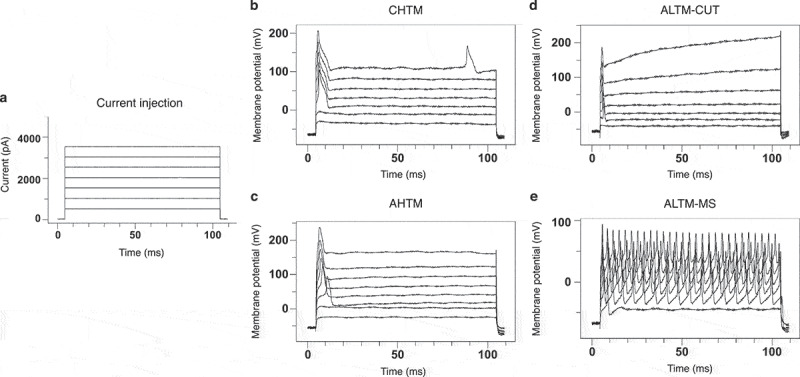
Soma excitability thresholds of sensory DRG neurons were determined by evoked action potentials in the soma of sensory DRG neurons induced using stimulation by direct injection of depolarizing current. Representative recordings show the intracellular current injection pulses with threshold and repetitive charges of evoked action potentials in different types of mechanoreceptor neurons in CIBP sham male rats

Action potential responses to intracellular depolarizing current pulse injection were tested in vivo to determine the soma excitability thresholds in sensory neurons at the DRG of model animals. Representative recording images show the multiple injected current stimuli (Figure 5a) of 100 ms each delivered between 500 to 4000 pA in increments of 500 pA and the characteristic evoked action potentials in the mechanoreceptor neuron types evaluated in this study, including CHTM (Figure 5b), AHTM (Figure 5c), and both CUT and MS ALTM neurons (ALTM-CUT: Figure 5d; ALTM-MS: Figure 5e). All CIBP model animals were tested following the conclusion of behavioral data collection at week 3.

There were no differences in ipsilateral limb weight bearing as a percentage of animal body weight as measured by dynamic weight bearing between PRO, PRE, and vehicle treatment groups of (a) male and (d) female CIBP model rats at any time point post model induction. (b) No treatment groups of male rats showed sustained maintenance of normal weight bearing past week 2 relative to baseline measurements. (e) PRE- and PRO-treated female rats also showed decreased weight bearing at week 3; however, vehicle-treated rats were not different from baseline measurements.At week 3 endpoint, all treatment groups of (c) male rats showed significantly decreased ipsilateral limb weight bearing relative to baseline and to sham controls, and PRE-treated rats were significantly lower than PRO-treated rats. At endpoint, (f) female PRE- and PRO-treated groups were significantly decreased from baseline and sham; however, vehicle-treated rats were significantly decreased from baseline measurements only.Data represent the mean ± SEM from male SD rats (PRE, *n* = 6; PRO, *n* = 5; vehicle, *n* = 5; sham, *n* = 5) and female SD rats (PRE, *n* = 6; PRO, *n* = 4; vehicle, *n* = 6; sham, *n* = 7). Only animals with verified tumor development were included in results shown. Differences between treatment groups over time (a, d) are compared to vehicle control by repeated measures two-way ANOVA with post hoc Dunnett’s test (#*P* < 0.05, PRE vs. vehicle; †*P* < .05, PRO vs. vehicle). Differences within treatment groups relative to baseline measurements (b, e) are compared by repeated measures two-way ANOVA with post hoc Dunnett’s test (*P < 0.05). Differences between treatment groups at endpoint (c, f) are compared by one-way ANOVA with post hoc Tukey’s test. Different letters (a, b, c) represent differences between groups (*P* < 0.05).

To quantify soma excitability, the threshold of depolarizing current pulses injected into the soma was determined. This was achieved by applying current injections of 100 ms each (a), delivered with an amplitude of 500 to 4000 pA with increments of 500 pA (*X*-axis: time [ms]; *Y*-axis: current [pA]). Representative raw recordings show the threshold and repetitive charges of APs (action potentials) evoked by intracellular current injection in the mechanoreceptor neurons evaluated in this study, including (b) C-type high-threshold mechanosensitive fibers (CHTM), (c) Aβ-type high-threshold mechanosensitive fibers (AHTM), (d) Aβ-low-threshold mechanosensitive fibers–cutaneous (ALTM-CUT), and (e) Aβ-low-threshold mechanosensitive fibers–muscle spindle (ALTM-MS). Discharge was evoked by injecting a series of depolarizing current pulses into DRG soma through the recording electrode (*X*-axis: time [ms]; *Y*-axis: voltage [mV]). All representative images here are from male CIBP model sham negative control rats.

**Figure 6.**
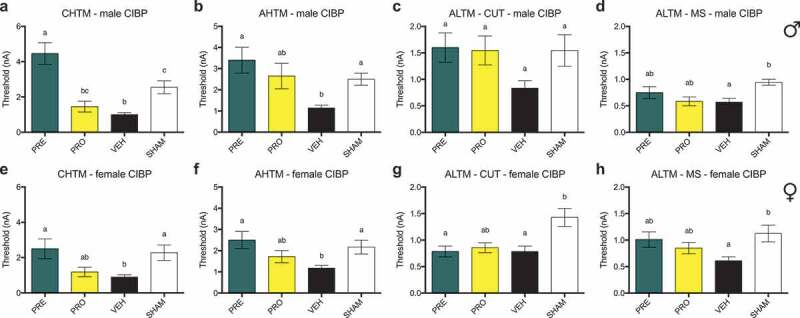
All types of sensory fibers tested demonstrate reduced excitability thresholds in vehicle-treated male and female cancer models. In high-threshold mechanosensitive fibers, PRE-treated animals had excitability thresholds equivalent to those of sham controls. Low-threshold mechanosensitive fibers were unaffected by treatment in both sexes

Current activation thresholds measured in nanoamps of CHTM neurons (Figure 6a) were significantly decreased in vehicle-treated male CIBP rats relative to sham control rats. Treatment with PRE significantly increased activation thresholds of CHTM above both vehicle and sham groups, whereas PRO-treated animals showed no differences in CHTM threshold from either vehicle or sham groups. Activation thresholds of AHTM neurons (Figure 6b) were significantly decreased in vehicle-treated male CIBP rats relative to sham. Treatment with PRE significantly increased activation thresholds of AHTM above vehicle, whereas PRO-treated animals showed no differences from either vehicle or sham groups. There were no significant differences in activation thresholds between any groups of ALTM-CUT neurons in males (Figure 6c). The activation threshold of ALTM-MS neurons (Figure 6d) in the vehicle-treated group was significantly decreased relative to sham, whereas both PRE- and PRO-treated ALTM-MS neurons were not significantly different from either vehicle-treated or sham male rats.

Current activation thresholds were recorded at endpoint (post week 3) in response to intracellular current injection at the DRG soma. Activation thresholds of CHTM neurons in PRE-treated (a) male and (e) female rats were increased relative to vehicle. PRO-treated rats were not different from either vehicle or sham. PRE treatment also significantly increased activation thresholds of AHTM relative to vehicle in both (b) male and (f) female rats. PRO-treated rats were not different from vehicle or sham. There were (c) no significant differences in activation threshold between any groups of ALTM-CUT neurons in male rats and (g) no effects of treatment with PRE or PRO on ALTM-CUT thresholds in female rats. In both (d) males and (h) females, the activation thresholds of ALTM-MS neurons in PRE- and PRO-treated rats were not significantly different from either vehicle-treated or sham groups.Data represent the mean ± SEM from male (upper panel) SD rat DRG neurons (CHTM: PRE, *n* = 13; PRO, *n* = 11; vehicle, *n* = 15; sham, *n* = 10; AHTM: PRE, *n* = 10; PRO, *n* = 10; vehicle, *n* = 14; sham, *n* = 11; ALTM-CUT: PRE, *n* = 15; PRO, *n* = 11; vehicle, *n* = 9; sham, *n* = 11; ALTM-MS: PRE, *n* = 10; PRO, *n* = 6; vehicle, *n* = 7; sham, *n* = 9) and female (lower panel) SD rat DRG neurons (CHTM: PRE, *n* = 8; PRO, *n* = 8; vehicle, *n* = 15; sham, *n* = 9; AHTM: PRE, *n* = 9; PRO, *n* = 9; vehicle, *n* = 14; sham, *n* = 12; ALTM-CUT: PRE, *n* = 10; PRO, *n* = 10; vehicle, *n* = 9; sham, *n* = 9; ALTM-MS: PRE, *n* = 10; PRO, *n* = 10; vehicle, *n* = 9; sham, *n* = 8). Differences between treatment groups are compared by Kruskal-Wallis test with post hoc Dunnett’s multiple comparison test. Different letters (a, b, c) represent differences between groups (*P* < 0.05). Abbreviations are as indicated in Figure 5.

In female CIBP model rats, the activation thresholds of CHTM neurons (Figure 6e) in the vehicle-treated group were significantly decreased relative to sham. Similar to males, PRE-treated females had significantly increased activation thresholds of CHTM from vehicle, but they did not significantly differ from PRO-treated or sham females. PRO-treated female rats showed no significant differences from either vehicle or sham CHTM. The activation thresholds of AHTM neurons in female rats (Figure 6f) were significantly decreased in vehicle-treated animals relative to sham. Activation thresholds of AHTM in PRE-treated female rats were higher than vehicle but not different from sham, whereas PRO-treated females showed no differences in AHTM threshold from either vehicle or sham groups. The changes in AHTM neurons showed equivalent patterns in both male and female rats. There was no evidence of treatment effects on the activation threshold of ALTM-CUT neurons in female rats (Figure 6g). Both PRE- and vehicle-treated groups showed reduced thresholds relative to sham control, and PRO-treated rats were not different from any group. The activation threshold of ALTM-MS neurons (Figure 6h) in vehicle-treated females was significantly decreased relative to sham and, similar to males, both PRE- and PRO-treated ALTM-MS neuron thresholds were not significantly different from either vehicle-treated or sham female rats.

### Two-week treatment with pregabalin, progesterone, or a combination induces large and sustained recoveries to baseline in ipsilateral paw withdrawal threshold in male rat models of neuropathic pain, whereas treated female rat models show increases in withdrawal thresholds relative to vehicle-treated rats but not to baseline thresholds

**Figure 7.**
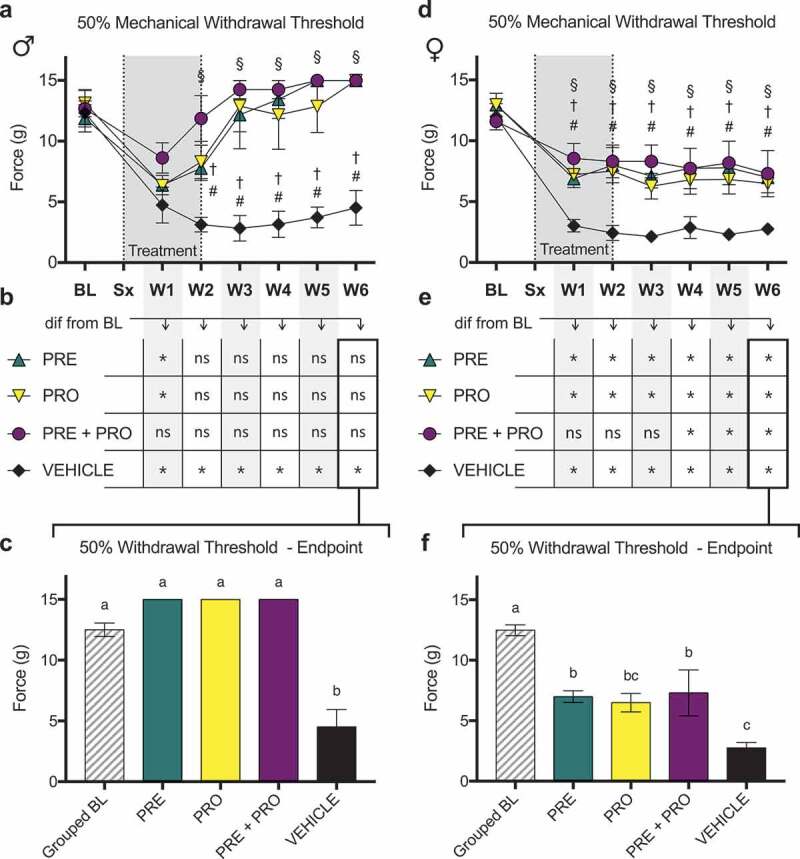
Male rat models of sciatic cuff–induced neuropathic pain treated with pregabalin, progesterone, or a combination showed large and sustained differences from vehicle-treated rats and recoveries to baseline ipsilateral paw withdrawal thresholds. Pregabalin-, progesterone-, and combination-treated female rat models also showed differences relative to vehicle-treated rats but did not show recoveries to baseline behavior

All treatment groups of male NEP model rats (Figure 7a) showed an initial decrease in 50% mechanical paw withdrawal threshold in the ipsilateral limb after model induction followed by a robust recovery where all treatment groups were increased from vehicle-treated rats at week 2 and all later time points. PRO and PRE treatment groups showed sustained recoveries to withdrawal thresholds no different from their respective baseline measurements (Figure 7b) by week 2. PRE+PRO combination–treated animals did not decline at any point to levels different from their baseline thresholds. In contrast, and characteristic of the sciatic cuff model, withdrawal thresholds of vehicle-treated animals remained significantly decreased from baseline at every postsurgical time point with no evidence of recovery. At week 6 endpoint (Figure 7c), paw withdrawal thresholds of all male treatment groups were not different from grouped baseline thresholds, and all treatment groups showed significantly increased thresholds relative to vehicle treatment. All groups of female NEP model rats also showed an initial decrease in 50% mechanical paw withdrawal threshold in the ipsilateral limb after model induction (Figure 7d); however, all treatment groups retained a consistently higher 50% paw withdrawal threshold than vehicle-treated rats at all time points post model induction. Unlike males, female treatment groups did not recover to withdrawal thresholds equivalent to their respective baseline measurements (Figure 7e) at any point following treatment initiation. PRE and PRO treatment groups remained lower than their respective baseline withdrawal thresholds at all postsurgical time points, and PRE+PRO combination treatment animals initially were no different from baseline but became different at week 4 and later. At endpoint (Figure 7f), there were no differences between treatment groups of female rats and all showed significantly decreased paw withdrawal thresholds relative to baseline. PRE and PRE+PRO combination–treated animals had higher withdrawal thresholds than vehicle, whereas PRO- and vehicle-treated animals were not different.

### Treatment with PRE prevents reduction in excitability thresholds in AHTM and ALTM-MS fibers in male rats, whereas all other types of sensory fibers tested demonstrate no changes in excitability thresholds in both sexes

**Figure 8.**
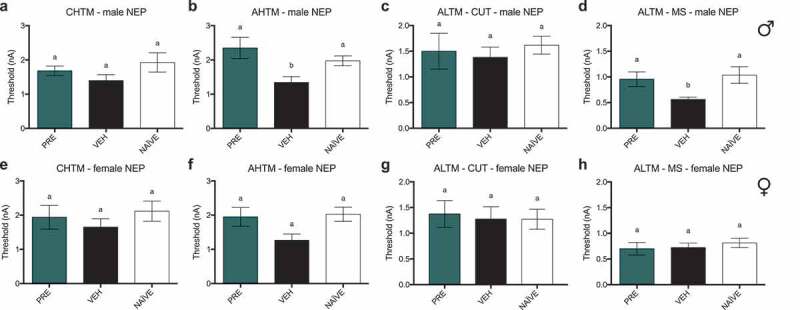
PRE-treated male NEP model SD rats showed current excitability thresholds in AHTM and ALTM-MS fibers that were significantly higher than vehicle-treated controls and equivalent to naïve rats. There were no differences in excitability threshold in any other fibers in males and females

In male NEP model rats, there were no differences between any groups in the current activation thresholds of CHTM neurons (Figure 8a). Activation thresholds of AHTM neurons (Figure 8b) were significantly decreased in vehicle-treated male NEP rats relative to both naïve and PRE-treated rats. There were also no differences in activation threshold between groups of ALTM-CUT neurons (Figure 8c), whereas the activation thresholds of ALTM-MS neurons (Figure 8d) were significantly decreased in vehicle-treated male NEP rats relative to both naïve and PRE-treated rats, which had thresholds equivalent to each other. In female NEP model SD rats, there were no differences between any groups in the current activation thresholds of all types of neurons tested: CHTM (Figure 8e), AHTM (Figure 8f) ALTM-CUT (Figure 8g), and ALTM-MS (Figure 8h) neurons.

### Female immunocompromised rat models of sciatic cuff–induced neuropathic pain treated with pregabalin and progesterone do not show sustained differences in ipsilateral 50% paw withdrawal thresholds from vehicle-treated rats or recoveries to baseline threshold levels

**Figure 9.**
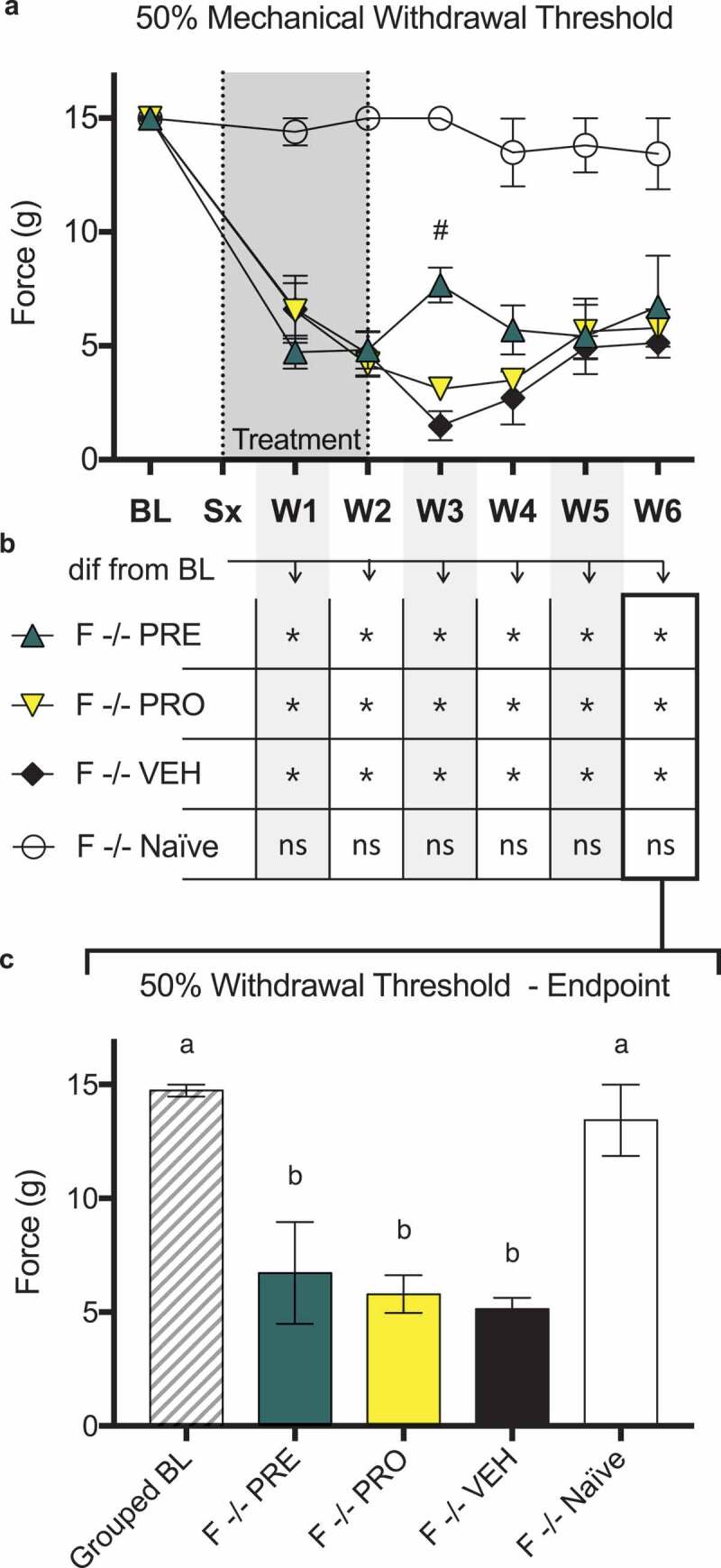
Female immunocompromised rat models of sciatic cuff–induced neuropathic pain treated with pregabalin and progesterone did not show sustained differences from vehicle-treated rats or recoveries to baseline ipsilateral paw withdrawal thresholds

There were no differences in 50% mechanical paw withdrawal threshold in the ipsilateral limb between PRO, PRE, and vehicle treatment groups of female RNU^−/−^ sciatic cuff–induced neuropathic pain model rats at any time point post model induction excluding week 3 as measured by testing with von Frey filaments (Figure 9a). At week 3, PRE-treated rats showed a significantly higher threshold than vehicle-treated rats; however, this difference was not sustained in later weeks.

Male and female models of sciatic cuff–induced NEP showed increased 50% mechanical paw withdrawal thresholds in response to treatment with pregabalin, progesterone, or a combination. (a) All treatment groups of male NEP model rats showed an initial decrease in 50% mechanical paw withdrawal threshold in the ipsilateral limb after model induction, followed by a robust recovery where all treatment groups were different from vehicle by week 2. (b) PRO and PRE treatment groups showed sustained recoveries to withdrawal thresholds no different from their respective baseline measurements by week 2. PRE+PRO combination–treated animals did not decline at any point to levels different from baseline. In contrast, withdrawal thresholds of vehicle-treated animals remained significantly decreased from baseline at every postsurgical timepoint. (c) At endpoint, paw withdrawal thresholds of all male treatment groups were not different from grouped baseline and were significantly increased relative to vehicle. (d) All groups of female NEP model rats also showed an initial decrease in withdrawal thresholds; however, all treatment groups remained higher than vehicle at all time points post model induction. (e) Unlike males, female treatment groups did not recover to baseline levels at any point following treatment. PRO and PRE treatment groups remained lower than their respective baseline withdrawal thresholds at all postsurgical time points, and PRE+PRO combination treatment animals initially were no different from baseline but became different at week 4 and later. (f) At endpoint, there were no differences between treatment groups and all showed significantly decreased withdrawal thresholds relative to baseline. PRE- and PRE+PRO combination–treated animals had higher withdrawal thresholds than vehicle, whereas PRO- and vehicle-treated animals were not different.Data represent the mean ± SEM from *n* = 4 male SD rats/group and female SD rats (PRE, *n* = 6. PRO, *n* = 6. PRE+PRO, *n* = 4. vehicle, *n* = 4). Differences between treatment groups over time (a, d) are compared to vehicle control by repeated measures two-way ANOVA with post hoc Dunnett’s test (#*P* < 0.05, PRE vs. vehicle; †*P* < 0.05, PRO vs. vehicle; §*P* < 0.05, PRE+PRO vs. vehicle). Differences within treatment groups relative to baseline measurements (b, e) are compared by repeated measures two-way ANOVA with post hoc Dunnett’s test (*P < 0.05). Differences between treatment groups at endpoint (c, f) are compared by one-way ANOVA with post hoc Tukey’s test. Different letters (a, b, c) represent differences between groups (*P* < 0.05).

Current activation thresholds awere recorded at endpoint (post week 6) in response to intracellular current injection at the DRG soma. There were no differences between any groups in the activation thresholds of CHTM neurons in (a) male and (e) female NEP model SD rats. (b) Activation thresholds of AHTM neurons in in PRE-treated male NEP rats were significantly higher than vehicle-treated neurons, which were significantly decreased relative to naïve. There were no differences in activation threshold between groups in AHTM neurons of female NEP rats. There were no significant differences in activation threshold between any groups of ALTM-CUT neurons in both (c) males and (g) females. Activation thresholds of ALTM-MS neurons in (d) PRE-treated male and (h) female NEP rats were significantly increased relative to vehicle in both groups.Data represent the mean ± SEM from male (upper panel) SD rat DRG neurons (CHTM: PRE, *n* = 11; vehicle, *n* = 15; naïve, *n* = 13; AHTM: PRE, *n* = 10; vehicle, *n* = 16; naïve, *n* = 20; ALTM-CUT: PRE, *n* = 10; vehicle, *n* = 17; naïve, *n* = 17; ALTM-MS: PRE, *n* = 11; vehicle, *n* = 16; naïve, *n* = 14) and female (lower panel) SD rat DRG neurons (CHTM: PRE, *n* = 8; vehicle, *n* = 13; naïve, *n* = 13; AHTM: PRE, *n* = 10; vehicle, *n* = 15; naïve, *n* = 19; ALTM-CUT: PRE, *n* = 8; vehicle, *n* = 9; naïve, *n* = 11; ALTM-MS: PRE, *n* = 5; vehicle, *n* = 9; naïve, *n* = 8). Differences between treatment groups are compared by Kruskal-Wallis test with post hoc Dunnett’s multiple comparison test. Different letters (a, b) represent differences between groups (*P* < 0.05). Abbreviations are as indicated in Figure 5.

(a) There were no differences in 50% mechanical paw withdrawal threshold in the ipsilateral limb between PRO, PRE, and vehicle treatment groups of female RNU^−/−^ sciatic cuff–induced neuropathic pain model rats at any time point post model induction excluding week 3 as measured by testing with von Frey filaments. At week 3, PRE-treated rats showed a significantly higher threshold than vehicle-treated rats; however, this difference was not sustained in later weeks.All model groups showed reduced thresholds relative to naïve control animals in all postsurgical weeks (not marked on chart). (b) In addition, all treatment groups including vehicle showed significantly reduced withdrawal thresholds relative to baseline measurements at all postsurgical time points. Naïve control rats were not different from baseline at any week. (c) At endpoint there were no differences between treatment groups and all showed significantly decreased paw withdrawal thresholds relative to baseline and to naïve control.Data represent the mean ± SEM from female RNU^−/−^ rats (PRE, *n* = 5; PRO, *n* = 5; vehicle, *n* = 4; naïve *n* = 3). (a) Differences between treatment groups over time are compared to vehicle control by repeated measures two-way ANOVA with post hoc Dunnett’s test (#*P* < 0.05, PRE vs. vehicle). (b) Differences within treatment groups relative to baseline measurements are compared by repeated measures two-way ANOVA with post hoc Dunnett’s test (*P < 0.05). (c) Differences between treatment groups at endpoint were compared by one-way ANOVA with post hoc Tukey’s test. Different letters (a, b) represent differences between groups (*P* < 0.05).

All model groups showed reduced thresholds relative to naïve control animals in all postsurgical weeks (not marked on chart; Figure 9b). In addition, all treatment groups including vehicle showed significantly reduced withdrawal thresholds relative to baseline measurements at all postsurgical time points. Naïve control rats were not different from baseline at any week (Figure 9c). At endpoint there were no differences between treatment groups and all showed significantly decreased paw withdrawal thresholds relative to baseline and to naïve control.

### No thresholds in any fiber types show differences from naïve or vehicle in response to treatment with PRE

**Figure 10.**
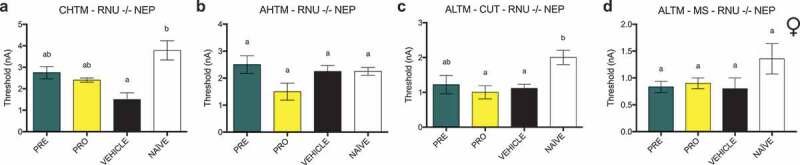
Immunocompromised female rat models of NEP showed decreases in the excitability thresholds of both CHTM and ALTM-CUT fibers. PRE treatment prevented this decrease relative to naïve animals, but these thresholds were not significantly different from vehicle

In athymic female RNU^−/−^ models of NEP, the current activation threshold of CHTM neurons (Figure 10a) was decreased in the vehicle-treated group relative to naïve RNU^−/−^ female rats. There were no significant differences in thresholds of CHTM neurons between both PRE- and PRO-treated groups and either vehicle or naïve rats. Activation thresholds of AHTM (Figure 10b) and ALTM-MS (Figure 10d) neurons showed no significant differences between any groups. Activation thresholds of ALTM-CUT neurons (Figure 10c) were significantly decreased from naïve in both the vehicle-treated and PRO-treated groups of female RNU NEP model rats. PRE-treated rats showed no differences in threshold from both either vehicle-treated or naïve rats.

### Male but not female rat models of sciatic cuff–induced neuropathic pain show an acute increase in ipsilateral paw withdrawal thresholds 1 h posttreatment with pregabalin, progesterone, or a combination at postsurgical week 1

**Figure 11.**
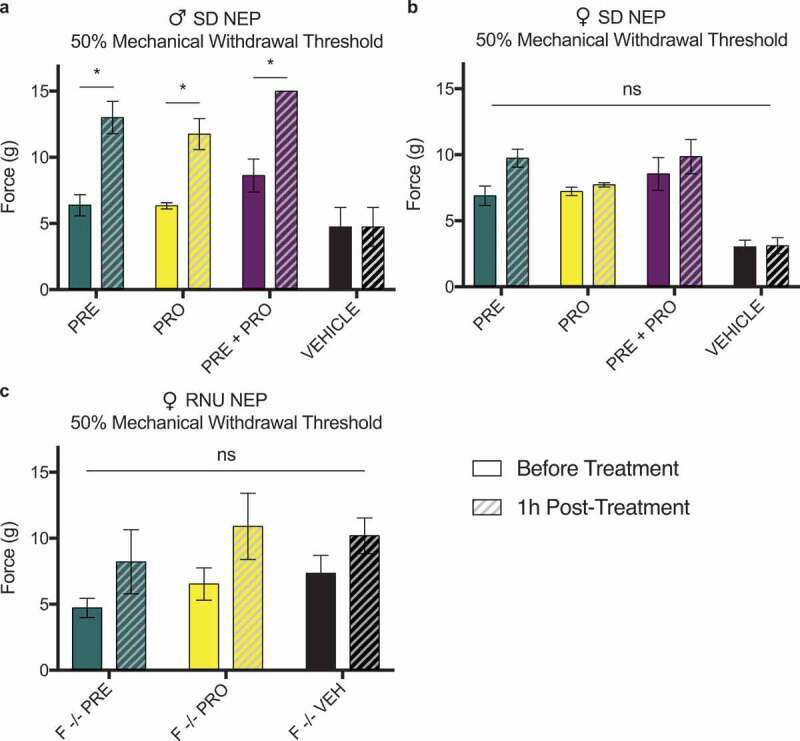
Male rat models of sciatic cuff–induced neuropathic pain showed an increase in ipsilateral paw withdrawal thresholds 1 h posttreatment with pregabalin, progesterone, or a combination at postsurgical week 1. This acute response to treatment was not seen in female immunocompetent or immunocompromised neuropathic pain model animals

All NEP model animals were measured at week 1 pretreatment and 1 h posttreatments. Male SD rat models of sciatic cuff–induced neuropathic pain showed an acute response to treatment with PRE, PRO, and PRE+PRO combination as an increase in 50% mechanical paw withdrawal threshold as measured by testing with von Frey filaments. Withdrawal thresholds of vehicle-treated animals did not change following treatment. No acute responses to treatment were seen in any groups of female immunocompetent SD rats (Figure 11b) or immunocompromised RNU^−/−^ neuropathic pain model animals (Figure 11c).

Current activation thresholds were recorded in athymic female RNU^−/−^ models of NEP at endpoint (post week 6) in response to intracellular current injection at the DRG soma. (a) Activation thresholds of CHTM neurons were decreased in the vehicle-treated group relative to naïve; however, there were no significant differences in the thresholds of CHTM neurons between both PRE- and PRO-treated groups and either vehicle or naïve rats. (b) There were no significant differences in activation threshold between any groups of AHTM neurons. (c) Activation thresholds of ALTM-CUT neurons were significantly decreased from naïve in both the vehicle-treated and PRO-treated groups. PRE-treated rats showed no differences in ALTM-CUT threshold from both vehicle-treated and naïve rats. (d) Activation thresholds of ALTM-MS neurons were not different between groups.Data represent the mean ± SEM from female RNU^−/−^ rat DRG neurons (CHTM: PRE, *n* = 8; PRO, *n* = 5; vehicle, *n* = 6; naïve, *n* = 7; AHTM: PRE, *n* = 7; PRO, *n* = 6; vehicle, *n* = 6; naïve, *n* = 4; ALTM-CUT: PRE, *n* = 9; PRO, *n* = 7; vehicle, *n* = 13; naïve, *n* = 15; ALTM-MS: PRE, *n* = 6; PRO, *n* = 5; vehicle, *n* = 5; naïve, *n* = 7). Differences between treatment groups are compared by Kruskal-Wallis test with post hoc Dunn’s multiple comparison test. Different letters (a, b) represent differences between groups (*P* < 0.05). Abbreviations are as indicated in Figure 5.

All NEP model animals were measured at week 1 pretreatment and 1 h posttreatments. (a) Male SD rat models of sciatic cuff–induced neuropathic pain showed an acute response to treatment with pregabalin, progesterone, or a combination as an increase in 50% mechanical paw withdrawal threshold as measured by testing with von Frey filaments. Withdrawal thresholds of vehicle-treated animals did not change following treatment. No acute responses to treatment were seen in any treatment groups of (b) female immunocompetent SD rats or (c) immunocompromised RNU^−/−^ neuropathic pain model animals.Data represent the mean ± SEM from *n* = 4 male SD rats/group and female SD rats (PRE, *n* = 6; PRO, *n* = 6; PRE+PRO, *n* = 4; vehicle, *n* = 4) and female RNU^−/−^ rats (PRE, *n* = 5; PRO, *n* = 5; vehicle, *n* = 4; naïve, *n* = 3). Comparisons within treatment groups are by multiple *t*-tests (*P < 0.05).

## Discussion

This study was designed to assess the effects of PRE and PRO on the development of chronic hypersensitivity in CIBP and NEP animal models in both male and female groups. We present for the first time that these treatments, administered as repeated injections during the early phases of NEP and CIBP development, promoted the robust recovery of mechanical hypersensitivity in male NEP rats, partial recovery of female NEP and female CIBP rats, and no apparent effects on both male CIBP and female immunocompromised NEP models. These results add to the evidence indicative of differing mechanisms of pain generation in CIBP and NEP states and to the evidence of sexual divergence in both the mechanisms of chronic pain itself and in the response to pain therapeutics.

The behavioral results of this study suggest that PRE and PRO have efficacy in treating male and female NEP rats, and electrophysiological data support this for PRE-treated animals, showing excitation thresholds in nociceptive neurons equivalent to naïve. However, CIBP model male animals show no behavioral response to PRE or PRO, and female CIBP rats show only limited evidence of a delay in the onset of hypersensitivity with both treatments. Our previous investigation of the intracellular electrophysiological characteristics of sensory neurons in this CIBP model indicated that there are both nociceptive and neuropathic components of the cancer-induced pain state. These include reduced signaling thresholds of ALTM-MS neurons in both vehicle-treated NEP and CIBP model rats relative to control and morphological changes at the spinal cord indicative of a possible role for ALTM fibers in the generation or maintenance of the neuropathic component of CIBP.^34^ In this study, both CHTM and AHTM nociceptive neurons and ALTM-CUT and ALTM-MS nonnociceptive neurons showed significant changes in CIBP models, and treatment with PRE induced the recovery of nociceptive neuron excitation thresholds to sham model levels in both sexes. There were no differences in treated ALTM neurons from vehicle control. In NEP male and female model animals, we have observed that CHTM and ALTM-CUT neurons did not change in any groups relative to naïve and that reductions in the thresholds of AHTM and ALTM-MS neurons were prevented or reversed by treatment with PRE in male NEP animals and were not significantly changed in any groups of female NEP animals. Taken together, these results indicate that in contrast to the strong behavioral responses to treatment in NEP model rats, which showed normally nonnociceptive ALTM thresholds equivalent to naïve, the absent and muted behavioral response to treatment in in male and female CIBP rats, respectively, may be associated with decreased signaling thresholds in ALTM-CUT and ALTM-MS neurons.

In this study, normally nociceptive CHTM and AHTM neurons in both pain models showed decreased excitability after treatment with PRE. Treatment with PRO produced partial reductions in neuronal excitability thresholds in CIBP animals, although none were significantly different from either vehicle or sham controls. There is preclinical and clinical evidence of the efficacy of both drugs for conditions involving neuronal damage and neuropathy; however, the exact mechanisms of action are unclear. PRO has been shown to have neuroprotective properties, including protection from excitotoxic cell death, demyelination, and reduction of neuronal inflammation and edema, all of which can contribute to the generation of ectopic signaling,^11,35^ and has demonstrated some utility in treating chronic NEP in rat models in male rats.^8^ PRE has demonstrated effectiveness for NEP in animal models and in humans, leading to its use as a first-line clinical therapy for NEP; however, its effectiveness is inconsistent.^12,36^ Both PRO and PRE inhibit VGCCs, and though the exact mechanisms of the PRO and L-type VGCC interaction have not yet been elucidated, PRE, like gabapentin, binds at the α2-δ subunits of P/Q-, N-, and L-type Ca^2+^ channels.^11,16^ T- and N-type VGCCs are expressed on sensory afferent neurons,^37,38^ and it has been reported that the α2δ-1 subunit is upregulated in DRG neurons in several animal models of pain, and this is causally related to the onset of pain behavior.^39–41^ It also has been reported that pregabalin reduces the depolarization-induced calcium influx at nerve terminals, resulting in a reduction of the presynaptic release of excitatory neurotransmitters, including glutamate, substance P, and CGRP.^42,43^ VGCCs are also expressed on glial cells^44^; however, their expression and function are less well understood.

A limitation of this study is that our animal models developed over different durations, where our endpoint electrophysiological data were recorded at week 6 in all NEP models and at week 3 in all CIBP models. In addition, unlike the one-time nervous system damage of the cuff NEP model, our CIBP model reflects the progressive and often intractable nature of cancer pain in humans. CIBP is a conglomerate of multiple initiating factors, including a wide range of nociceptive and inflammatory stimuli, including mechanical distortion and pressure on host tissues, secreted inflammatory and nociceptive mediators, and neuropathy from tumor-initiated destruction and damage of nervous tissue and pathological growth of new and dysregulated sensory neurons.^45,46^ Electrophysiological measurements from week 3 endpoint either may not reflect a delay in the onset of this pain state or may not have allowed for an adequate and comparable duration of recovery.

Female CIBP rats treated with PRE and PRO showed a delay to the onset of mechanical hypersensitivity as measured by von Frey fibers. This delay was not reflected by measures of either weight bearing or limb use. Male CIBP model rats did not show behavioral responses to treatment. In NEP model rats, males demonstrated a strong response to drug treatment where PRE, PRO, and PRE+PRO treatment groups recovered to 50% mechanical thresholds equivalent to their baseline measurements by week 2. Although all NEP model female treatment groups remained at significantly higher withdrawal thresholds than vehicle-treated female controls throughout the experiment, no treatment groups recovered to baseline levels, in contrast to male rats. These sexually divergent effects in response to treatment are suggestive of mechanistic differences between male and female CIBP and NEP models. Substantial evidence indicates that sex differences in the behavioral responses to peripheral neuropathy in animal models may involve distinct hormonal and immune system pathways. After peripheral nerve injury, microglial–neuronal signaling in the spinal cord appears to mediate hypersensitivity in male mice, whereas in females, despite concurrent microglial proliferation, T cells infiltrate the spinal cord and maintain a hypersensitive state.^47–49^ There is evidence in cancer pain states, however, that microglia do play a role in the maintenance of pain in female rats.^50^ To investigate whether the sex differences observed in our NEP models involved the T cell–dependent signaling systems in the spinal cord, we applied the sciatic cuff model to female RNU^−/−^ athymic rats. Our hypothesis was that the lack of mature T cells would result in a chronic pain state with behavioral responses similar to those of immunocompetent male rats, as has been shown in other animal models of pain.^47^ It has also been demonstrated that T cell–deficient nude rats develop significantly reduced mechanical allodynia following chronic constriction injury compared with their heterozygous littermates.^51^ Our findings showed a behavioral difference between PRE- and vehicle-treated rats at only one time point, and that difference was not sustained. Female athymic NEP model rats in fact demonstrated more severe mechanical hyperalgesia in treatment groups than immunocompetent females, counter to our expectations, although this could reflect strain differences. Correspondingly, CHTM and ALTM-CUT neurons in vehicle-treated immunocompromised female NEP models showed increased excitability relative to naïve controls and were not recovered by treatment.

Sex differences were also apparent in the acute behavioral response measurements performed at week 1 in all NEP model animals. Male NEP rats of all treatment groups responded with an increase in mechanical withdrawal threshold measured 1 h after treatment. These increases were not apparent in vehicle-treated controls, and no groups were significantly different in female immunocompetent and immunocompromised animals.

These discrepancies in the response to treatment with PRE and PRO between sexes and immunocompromised animals indicate that the cellular immune response in the spinal cord of animal models of chronic pain is not a simple answer in this case. Inflammatory responses to injury also have adaptive functions enabling nerve repair involving interleukin-1 and tumor necrosis factor-α expression,^52^ and complete ablation of macrophages can result in severely impaired axon regeneration.^53^ It is possible that a partially beneficial immune response to chronic injury and NEP, as is the case in the sciatic cuff model, could be limited by the absence of T cells and by therapies that target them.

In conclusion, we show that the analgesic effects of PRE and PRO can promote recovery of tactile hypersensitivity in response to treatment initiated during the early phase of NEP development. These effects are sex dependent and strongest in male rat models of NEP. Female rat models of NEP show a limited response to PRE and PRO treatment. Female CIBP models also show a limited response to treatment, and males do not respond. These results indicate that sex may be an important consideration for the therapeutic utility of both PRE and PRO.
